# Circulating Tumor Cells (CTCs) Detected by RT-PCR and Its Prognostic Role in Gastric Cancer: A Meta-Analysis of Published Literature

**DOI:** 10.1371/journal.pone.0099259

**Published:** 2014-06-05

**Authors:** Shuyi Wang, Gang Zheng, Boran Cheng, Fangfang Chen, Zhenmeng Wang, Yuanyuan Chen, You Wang, Bin Xiong

**Affiliations:** 1 Department of Oncology, Zhongnan Hospital of Wuhan University, Hubei Key Laboratory of Tumor Biological Behaviors, Hubei Cancer Clinical Study Center, Wuhan, Hubei, P. R. China; 2 Department of General Surgery, The 5^th^ Hospital of Wuhan, Wuhan, Hubei, P. R. China; West German Cancer Center, Germany

## Abstract

**Objective:**

The prognostic significance of circulating tumor cells (CTCs) is controversial in gastric cancer (GC). We performed a meta-analysis of available studies to assess its prognostic value detected by RT-PCR for patients diagnosed with GC.

**Methods:**

EMBase, PubMed, Ovid, Web of Science, Cochrane library and Google Scholar database search was conducted on all studies reporting the outcomes of interest. The studies were set up according to the inclusion/exclusion criteria. Meta-analysis was performed by using a random-effects model; hazard ratio (HR), risk ratio (RR) and their 95% confidence intervals (95% CIs) were set as effect measures. The information about trial design, results from the data was independently extracted. Heterogeneity of the studies was tested for each pooled analysis.

**Results:**

Nineteen studies published matched the selection criteria and were included in this meta-analysis. CTCs positivity was significantly associated with poor relapse free survival (RFS) (HR 2.42, 95% CI: [1.94–3.02]; P<0.001) and poor overall survival (OS) (HR 2.42, 95% CI: [1.94–3.02]; P<0.001). CTCs positivity were also significantly associated with regional lymph nodes (RLNs) metastasis (RR 1.42, 95% CI: [1.20–1.68]; p<0.0001), depth of infiltration (RR 1.51, 95% CI: [1.27–1.79]; p<0.0001), vascular invasion (RR  = 1.43, 95% CI: [1.18–1.74], p = 0.0002) and TNM stage(I,II versus III) (RR 0.63, 95% CI [0.48–0.84]; p = 0.001).

**Conclusion:**

Preoperative CTCs positivity indicates poor prognosis in patients with gastric cancer, and associated with poor clinicopathological prognostic factors.

## Introduction

Globally, gastric cancer (GC) is the fourth most common cancer and is the second leading cause of cancer –related death [1]. In China, gastric cancer holds the third place of morbidity among digestive system cancers, due to the difficulties of early diagnosis, quantities of patients were diagnosed with GC until in its advanced stage; unfortunately, even after radical operation and adjuvant therapy, the 5-year overall survival (OS) of GC patient is relatively low (under 50%) [2]; over the past decade, therapy strategy of gastric cancer continuously changes but still fails to improve overall prognosis significantly, most of patients die because of distant metastasis and recurrence. Thus, in order to improve the clinical outcome of GC patients, we need new biomarkers that can help us to identify patients with high-risk of metastasis and pursue specific therapy strategy.

As is known to us, tumor metastasis consists a series of biological procedures, one important step is tumor cells disseminate into blood stream and circulate [3]; thus, to get more insights into metastasis cascade, studies of circulating tumor cells (CTCs) vigorously becomes one of hot academic topics. The concept of CTCs dated back to the study of Ashworth [4] in1869 and was demonstrated by Engell [5] in 1955 who proved the existence of these rare cells. There is a considerable body of evidence indicating that CTCs are shed from the primary tumor mass at a earliest stages of malignant progression [6]; these cells, circulating through the bloodstream, traveling to different tissues of body, are the main cause of overt metastases [7]. Nowadays, numerous studies have investigated the prognostic relevance of CTCs positivity of patients with breast cancer [8], colorectal cancer [9], and proved that CTCs could be a poor prognostic marker.

With regard to gastric cancer, although there are many studies designed to find out the relationship between CTCs and prognosis or other clinicopathologic parameters, the lack of statistical power together with their different study design and results limited the individual clinical value and the prognostic effect of CTCs positivity. Especially, the value of preoperative CTCs positivity in gastric cancer patients has not yet been clearly illustrated. Thus we performed a combined analysis of available studies that will provide a more precise estimate on the prognostic relevance of CTCs in patients with GC.

## Methods

### Literature Search

PubMed, Embase, Ovid, Web of Science, Google Scholar and Cochrane library data bases were systematically searched without time restrictions. Studies reporting on the molecular detection of CTCs and its effect on prognosis in gastric cancer were identified. The following key words were used: ‘‘Circulating tumor cells’’ or ‘‘CTCs’’, ”gastric cancer’’, “prognosis” and “PCR” were used as the key words. In order to prevent missing relevant publications, “related articles” function of Pubmed and Google Scholar were used to identify other potentially relevant publications. References of the articles were hand-searched for relevant articles, including review articles. Two reviewers (S.Y. Wang and G Zheng) independently screened and retrieved the literature list and, in the case of potentially relevant references, obtained the full articles; Cases of disagreement were resolved by discussing the title and abstract; Full-text articles (n = 39) were examined and 20 were excluded following the criteria below.

### Literature Screening Criteria

To be included in the analysis, studies had to match the following inclusion criteria: (1) any form of reverse transcription PCR (RT-PCR) used for the evaluation of the association between the putative markers of circulating tumor cells and either overall survival (OS), relapse-free survival (RFS), or prognostic factors of gastric cancer; (2) >20 analyzed patients and sufficient data to calculate a hazard ratio (HR) or a risk ratio(RR) with a 95% confidence interval (95% CI) as a comparable effect estimate; (3) samples used in these studies should be peripheral blood and should be collected before surgery; (4) exclusion of letters to the editor, reviews, and articles published in non-English language books or papers.

### Data Extraction

Two reviewers (S.Y. Wang and G Zheng) independently extracted the following data from each study: the year of publication, the first author’s surname, the number of cases and controls, the number of different clinical and pathological parameters, and the assessment methods of survival expression. Disagreements were resolved by discussion and were checked by a third investigator.

### Statistical Analysis

Statistical analysis were done with Review Manager (RevMan)(Version 5.2. Copenhagen: The Nordic Cochrane Centre, The Cochrane Collaboration, 2012). To statistically evaluate the prognostic effect of CTCs, we extracted Hazard Ratio (HR) and their associated standard errors on relapse free survival (RFS) and/or overall survival (OS) from the included studies. If the HRs and their associated standard errors, confidence intervals (CIs), or P values were not directly provided in the original articles, we approximated HRs according to the method described by Parmar et al [10]. By convention, a HR>1 implies a worse prognosis in the CTCs–positive group in comparison to negative group. We pooled the extracted HRs with the use of the generic inverse variance method available in the Review Manager. Because we expected interstudy heterogeneity, we applied a random effect model [11], because it is more conservative by creating a wider CI around the pooled HR than the fixed effect analysis model. When analyzed the association between CTCs and other parameters, Relative Risk (RR) was calculated, a RR>1 implied CTC-positive group was associated with a parameter. All data extractions were performed separately by SY Wang and G Zheng. Disagreements were resolved by discussion. Heterogeneity between studies was tested with the Q test and I^2^ statistic. We evaluated potential publication bias by a funnel plot, which was further examined by the Egger [12] and Begg’s test [13] using STATA software (Version 11.0, College Station, TX). And pooled analysis of the diagnostic accuracy of CTCs positivity was also calculated by STATA.

## Results

### Baseline Study Characteristics

**Figure 1:**
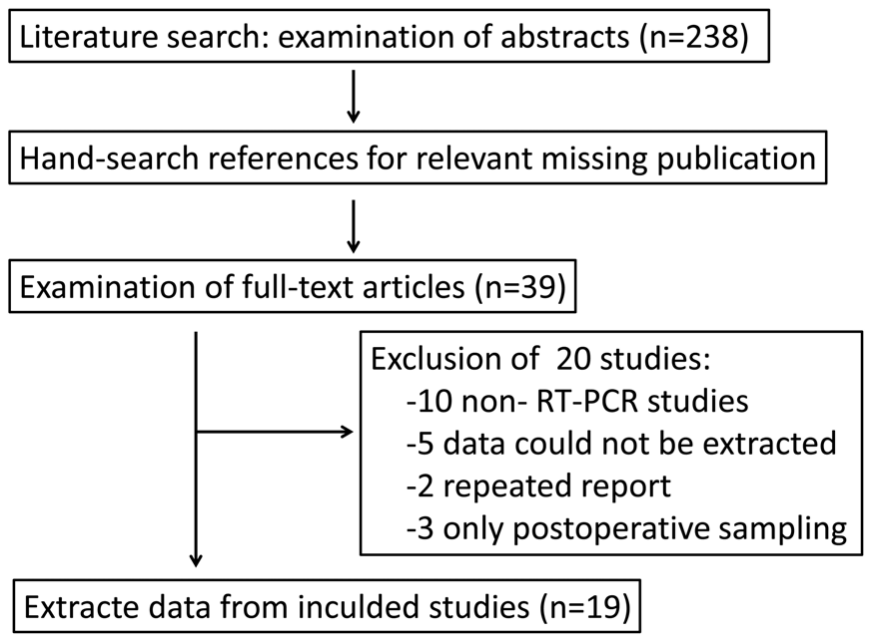
Flowchart of studies screening process.

The systematic literature search (Fig. 1) yielded a total of 19 studies [14]–[32] for final analysis. The studies were conducted in 6 countries (China, Germany, Japan, Korea and the United States) and published between 2000 and 2013. All 19 studies analyzing peripheral blood before surgery applied a molecular detection method (PCR, RT-PCR, or RT followed by quantitative PCR) of tumor cells, CEA mRNA was tested in 6 studies, and other genes were tested not more than 3 studies. The baseline characteristics of the included studies are summarized in (Table 1).

**Table 1:** Baseline characteristics of included studies for the meta-analyses.

First author	Year	Number of patients	RT-PCR detection method	Circulating tumor cell incidence	Cancer stages	Follow-up (months)	Outcomes measured
Majima	2000	52	CK19,CK20	5(9.6%)	I–IV	18	OS
Nishida	2000	41	CEA	12(29.3%)	I–IV	NR	NR
Miyazono	2001	57	CEA	21(36.8%)	I–IV	15(6–30)	RFS
Shin	2002	65	hTert	30(46.2%)	I–IV	NR	NR
		42	cMET	9(21.4%)			
Sumikura	2003	106	CEA	43(40.6%)	I–III	21(12–60)	RFS
Seo	2005	46	CEA	9(19.6%)	I–III	>6	RFS
Illert	2005	70	CK20	29(41.4%)	I–IV	20(1–57)	OS
Ikeguchi	2005	59	CEA	27(45.8%)	I–IV	20.1(2–31)	RFS
Wu	2006	42	Htert	26(61.9%)	I–IV	18(10–26)	RFS
			CK19	29(69%)			
			CK20	26(61.9%)			
			CEA	33(78.6%)			
Uen	2006	52	MUC1	37(71.2%)	I–IV	36	RFS
			c-MET	32(61.5%)			
Kosaka	2007	90	VEGFR-1	34(37.8%)	I–IV	9.8(4–24)	RFS
Koga	2008	69	CK19	8(11.6%)	I–IV	NR	OS
			CK20	10(14.5%)			
Yie	2008	55	Survivin	25(45.5%)	I–IV	36	RFS
Bertazza	2009	70	Survivin	69(98.6%)	I–IV	15(6–119)	OS
Kita	2009	846	uPAR	404(47.8%)	I–IV	NR	NR
Qiu	2010	123	CEA	45(36.6%)	I–IV	37(3–73.6)	RFS
Arigami	2011	95	B7-H3	48(50.5%)	I–IV	24(1–74)	OS
Cao	2011	98	Survivin	45(45.9%)	I–IV	47.5(36.5–56)	RFS
Agarami	2013	93	STCs	43(46.2%)	I–IV	25(1–74)	OS

**Foot note**: NR not reported, OS overall survival, RFS recurrence-free survival.

### Diagnostic Accuracy of CTCs Detection

To evaluate the overall test performance of included studies [14]–[17], [19], [21]–[32], we calculated the pooled diagnostic accuracy of CTCs detection. The combined sensitivity and specificity was 0.45 (95% CI: [0.34–0.57]) and 0.99 (95% CI: [0.96–1.00]) respectively (Figure S1), with significant heterogeneity (I^2^ = 91%, p<0.05 and I^2^ = 69.83%, p<0.05). Positive Likelihood Ratio (PLR) is 37.1 (95% CI: [11.7–118.1]), Negative Likelihood Ratio (NLR) was 0.55 (95% CI: [0.38–0.89]) (Figure S2). Combined diagnostic odds ratio was 67.08 (95% CI: [19.75–227.86]) (Figure S3) and the area under SROC curve was 0.93 (95% CI: [0.91–0.95] (Figure S4).

### Overall Analysis of Survival for Gastric Cancer Patients

**Figure 2:**
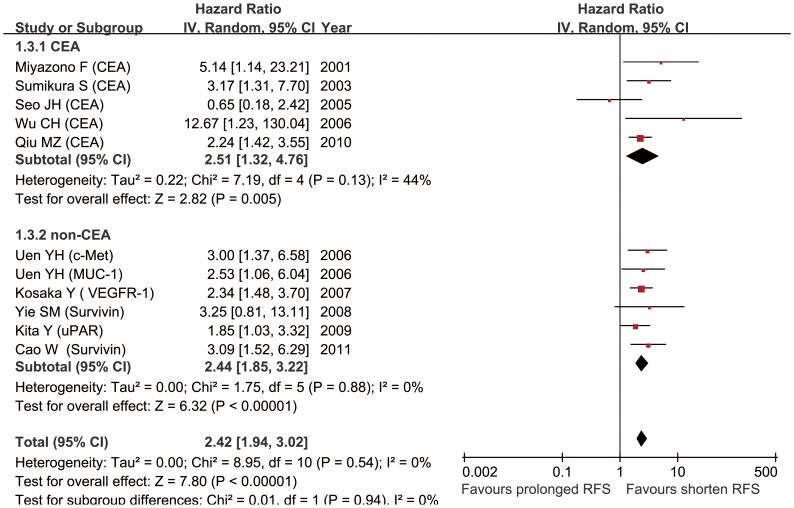
Summary estimates of hazard ratio (HR) for RFS. RFS, relapse-free survival.

Data on RFS were available in 10 studies [16], [18], [19], [22]–[24], [26], [28], [29], [31], the pooled analysis showed a prognostic effect of CTCs positivity (HR = 2.42, [95% CI: 1.94–3.02]; P<0.001) (Figure 2), with no between-study heterogeneity (I^2^ = 0%, p = 0.54). We also stratified studies of CEA-mRNA positive CTCs [16], [18], [19], [22], [29] for subgroup, pooled analysis suggested an association between poor RFS and CTCs positivity (HR = 2.51, 95% CI: [1.32–4.76], p<0.001%), and between-study heterogeneity was moderate (I^2^ = 44%, p = 0.13). Publication bias, tested by Egger’s test (p = 0.578) and funnel plot (Figure S5), was negligible for the pooled analysis of RFS.

**Figure 3:**
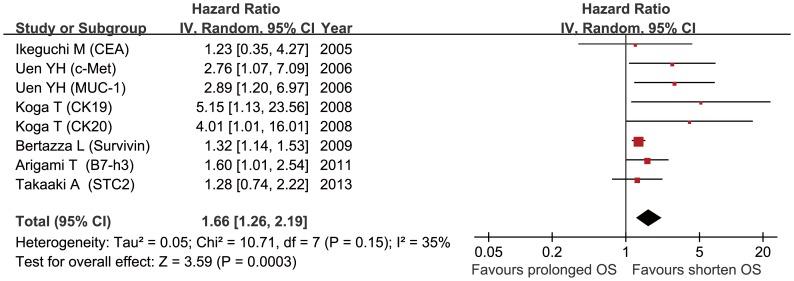
Summary estimates of hazard ratio (HR) for OS. OS, overall survival.

Pooled analysis of studies [21], [23], [25], [27], [30], [32] on OS showed that presence of CTCs was associated with poor OS (HR = 1.66, 95% CI: 1.26–2.19; p<0.001) (Figure 3), and the between-study heterogeneity (I^2^ = 35%, p = 0.15) was not significance. Egger’s test (p = 0.017) and funnel plot (Fig. S6) showed this combined analysis had publication bias.

### Correlation of Circulating Tumor Cells with Clinicopathologic Parameters

**Figure 4:**
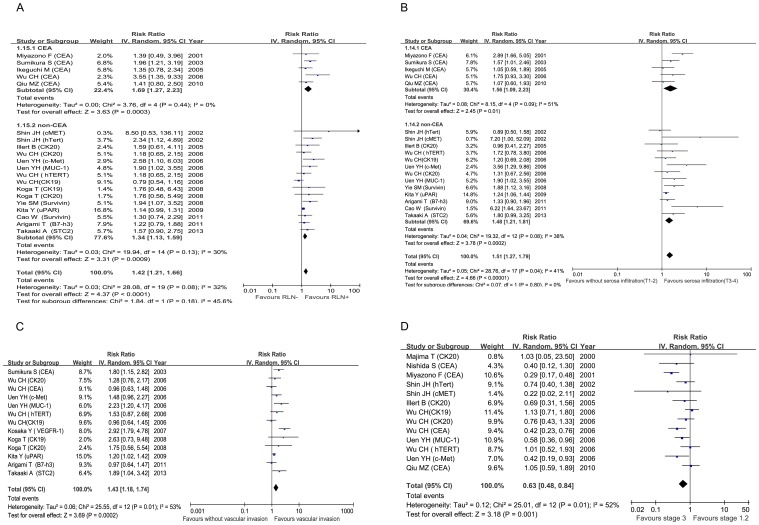
Summary estimates of risk ratio (RR) for RLNs metastasis (A), depth of infiltration (B), vascular invasion (C) and TNM stage (D) (Stage I,II vs Stage III) associated with CTCs positivity. RLNs, regional lymph nodes.

14 studies [16]–[18], [20]–[23], [25], [26], [28]–[32] were assessed the relationship between CTCs positivity and regional lymph nodes (RLNs) metastasis (RR = 1.42, 95% CI: [1.21–1.66]; p<0.0001) (Fig. 4A), with no significant between-study heterogeneity (I^2^ = 32%, p = 0.08), subgroup analysis showed that CEA-mRNA positive CTCs were associated with RLNs metastasis (RR = 1.69, 95%CI:[1.27–2.23]; p = 0.0003), and the between-study heterogeneity decreased (I^2^ = 0%, p = 0.44). Studies [16]–[18], [20]–[23], [26], [30]–[32] assessed by pooled analysis showed significant association of CTCs positivity with the depth of tumor infiltration (RR = 1.51, 95% CI: [1.27–1.79]; p<0.0001) (Fig. 4B), between-study heterogeneity was significant (I^2^ = 41%, p = 0.04), subgroup analysis showed that CEA-mRNA positive CTCs were associated with depth of tumor infiltration (RR = 1.56, 95% CI:[1.09–2.23], p = 0.01), with same between-study heterogeneity (I^2^ = 51%, p = 0.09). Vascular invasion[18], [22]–[25], [28], [30], [32] (RR  = 1.43, 95% CI: [1.18–1.74]; p = 0.0002) was associated with CTCs positivity (Fig. 4C), but the between-study heterogeneity was significant (I^2^ = 55%, p = 0.01).

Eight studies [14]–[17], [20], [22], [23], [29] reported the relationship between CTCs positivity and TNM stage, the overall positive rate of CTCs in stage I and II group was 36.7% compared with 56.6% of stage III group. Pooled analysis showed that CTCs positivity in stage III is greater than on stage I and II (RR 0.63, 95% CI 0.48–0.84; p = 0.001), with between-study heterogeneity (I^2^ = 52%, p = 0.01) as shown in Figure 4D. When pooled analysis [14]–[17], [20], [22], [23], [26], [29] was introduced to compare CTC positivity in stage I with stage II, the CTCs positivity was higher in stage II versus stage I (RR = 0.55; [95% CI 0.36–0.84], p = 0.005). However, when stage II and stage III groups were compared [15]–[17], [20], [22], [23], [29], data showed no statistically significant (RR = 0.87; [95% CI: 0.73–1.04], p = 0.93).

## Discussion

From the clinical perspective, the assessment of patients’ prognosis by CTCs detection in the PB can supply important prognostic information. Bizard et al [33] found that even a single CTC detected in 7.5 ml of blood was associated with the subsequent development of metastases, which means CTCs have strong potential of distant metastasis. Besides, CTCs detection, with the advantage of time- and cost- saving, appears comfortable for the patient and may be easily repeated as a monitoring tool. To date, encouraging results concerning an association between CTC positivity and metastatic progression in patients with metastatic breast [34], prostate [35], and colorectal [36] cancer have been recently published. However, there is currently very limited data on the clinical relevance of CTC positivity in GC patient, the results of our collective evaluation suggest that CTCs positivity in PB should indeed be considered as a prognostic marker.

During the process of our meta-analysis, we restrict sampling time and site for our design in order to minimize heterogeneity, but we still notice a certain degree of heterogeneity. Potential sources of heterogeneity may derive from differences in the detection protocol, types and numbers of target genes selection, standard of CTCs positivity, as well as in demographic or clinicopathologic data of included patients. In theory, postoperative CTCs status may be important and informative, it reflects the combined information of preoperative CTCs and intraoperative tumor cell release by surgical manipulation [37]. But the rapid apoptotic death of freshly shredded CTCs may release mass tumor gene or antigens because of the loss of survival microenvironment in the systemic circulation; this may lead to certain degree of detection bias. Sampling time is another important factor that interfere the prognositic value of CTCs positivity and leads to heterogeneity. Ikeguchi M et al. [21] studied the association between postoperative CTCs positivity and prognosis, they found that, if the blood samples were postoperatively collected within 48 hours, CTCs positive patients had better prognosis than CTCs negative ones. Thus, further studies of CTCs should take sampling time into consideration, evaluate and confirm the best sampling time. A further source for the observed heterogeneity may be the CTCs pool itself, it was consisted of heterogeneous population of cancer cells, within this population only a specific fraction had prognostic effect [38]. Furthermore, characterization of CTCs with breast cancer, gastric cancer, or colorectal cancer showed that only a minority of these cells express proliferation-associated markers, growth factor receptors, immune response antigens, adhesion molecules, and proteases or protease-associated proteins [39].In addition, tumor cells dissemination is an early event during distant metastasis, and random aberrations for metastasis-specific gene may be acquired after CTCs shedding into the blood circulation [40]. This model may explain the genomic and functional heterogeneity of CTCs.

There are some limitations of this meta-analysis. Firstly, limitations caused by the heterogeneity mentioned before and the inability to access primary data of the included studies. We addressed the issue of heterogeneity by a rigorous methodological approach that used the random-effects model for more conservative estimates. Prognostic factors of gastric cancer are complicated, our data for meta-analysis was from the included studies and primary data was hard to get, we were unable to exclude every possible confounding factors; approaches based on RT-PCR have high sensitivity for the detection of CTCs, but they cannot quantify the number of CTCs and lack biologic specificity [38]. Secondly, languages selection brings another bias, we have restricted our analysis to published studies written in English, other language such as Japanese, German were excluded based on language criteria. This may result in language bias leading to an overestimation of effect sizes [41]. Thirdly, we notice that certain degree of publication bias exists, especially in the pooled analysis for OS, one reason may be that studies reported positive results are much easier to be published and accessed; besides, studies introduced to pooled analysis have relatively small sample size. Although we were unable to conduct analyses considering certain potentially relevant factors, CTC positivity representing an indicator of poor prognosis in GC patients was consistently present in the pooled analysis; however, our results should be interpreted with caution and it requires more detailed and accurate data to verify.

In conclusion, our study based on available evidence supports the notion of a strong prognostic value of CTCs in the peripheral blood and relates to poor prognosis of GC. Identification of various methodological flaws and sources of heterogeneity in currently available prognostic factor studies could contribute to improve design and reporting of future prognostic and predictive factor studies. Our results also offer a hint that additional studies should use standardized testing method, optimized sampling time, complete analysis and report of results, or identification of certain cellular subgroup such as circulating stem-like cells [42]; in this way can we derive clearer and more accurate prognostic significance of CTCs in GC patients.

## Supporting Information

Figure S1 **Forest Plot for pooled analysis of SEN and SPE. SEN, sensitivity; SPE, specificity.**
(TIF)

Figure S2 **Forest Plot for pooled analysis of PLR and NLR. PLR, positive likelihood ratio; NLR, negative likelihood ratio.**
(TIF)

Figure S3 **Forest Plot for pooled analysis of DOR. DOR, diagnostic odds ratio.**
(TIF)

Figure S4 **Summary ROC curve with confidence and prediction regions of sensitivity and specificity.**
(TIF)

Figure S5 **Funnel plot for summary estimates of RFS. RFS, relapse-free survival.**
(TIF)

Figure S6 **Funnel plot for summary estimates of OS. OS, overall survival.**
(TIF)

CheckList S1 **PRISMA checklist.**
(DOC)
