# Supplementary figures and images for: Circulating Tumor Cells (CTCs) Detected by RT-PCR and Its Prognostic Role in Gastric Cancer: A Meta-Analysis of Published Literature
Source: PLoS One. 2014 Jun 5;9(6):e99259. doi: 10.1371/journal.pone.0099259 (PMC4047117; doi:10.1371/journal.pone.0099259)

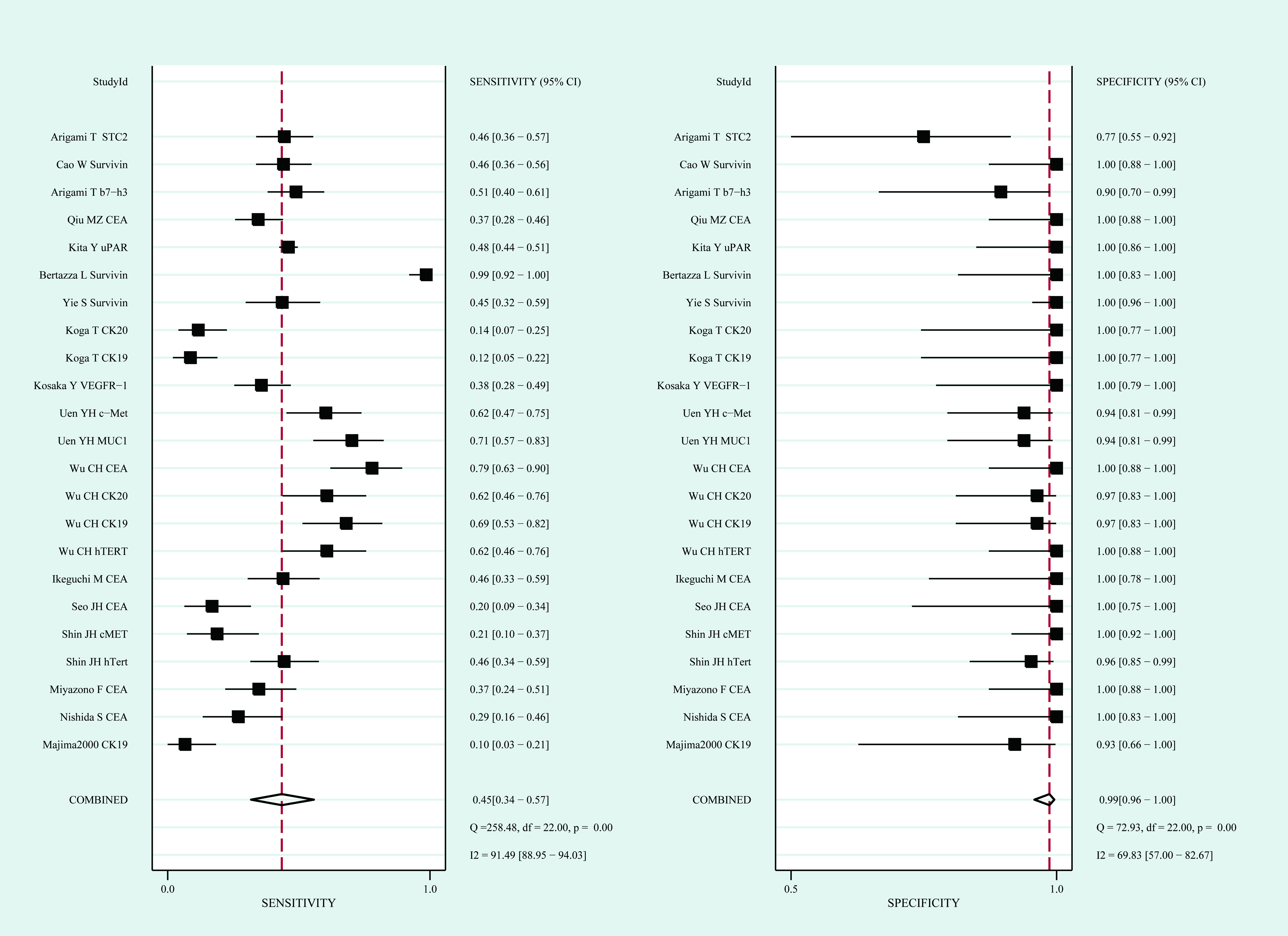

Supplement: Figure S1 — Forest Plot for pooled analysis of SEN and SPE. SEN, sensitivity; SPE, specificity. (TIF) [file pone.0099259.s001.tif]

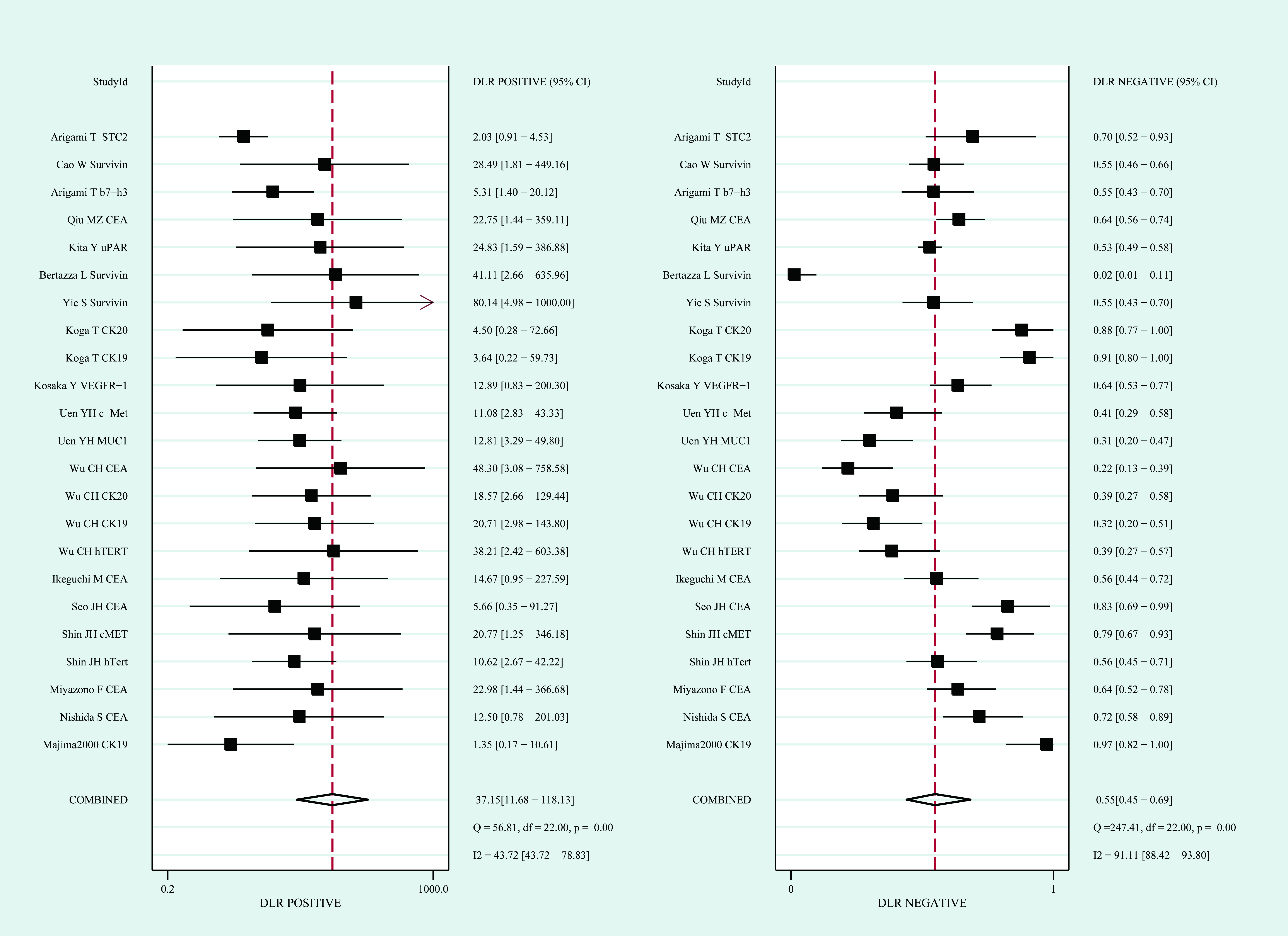

Supplement: Figure S2 — Forest Plot for pooled analysis of PLR and NLR. PLR, positive likelihood ratio; NLR, negative likelihood ratio. (TIF) [file pone.0099259.s002.tif]

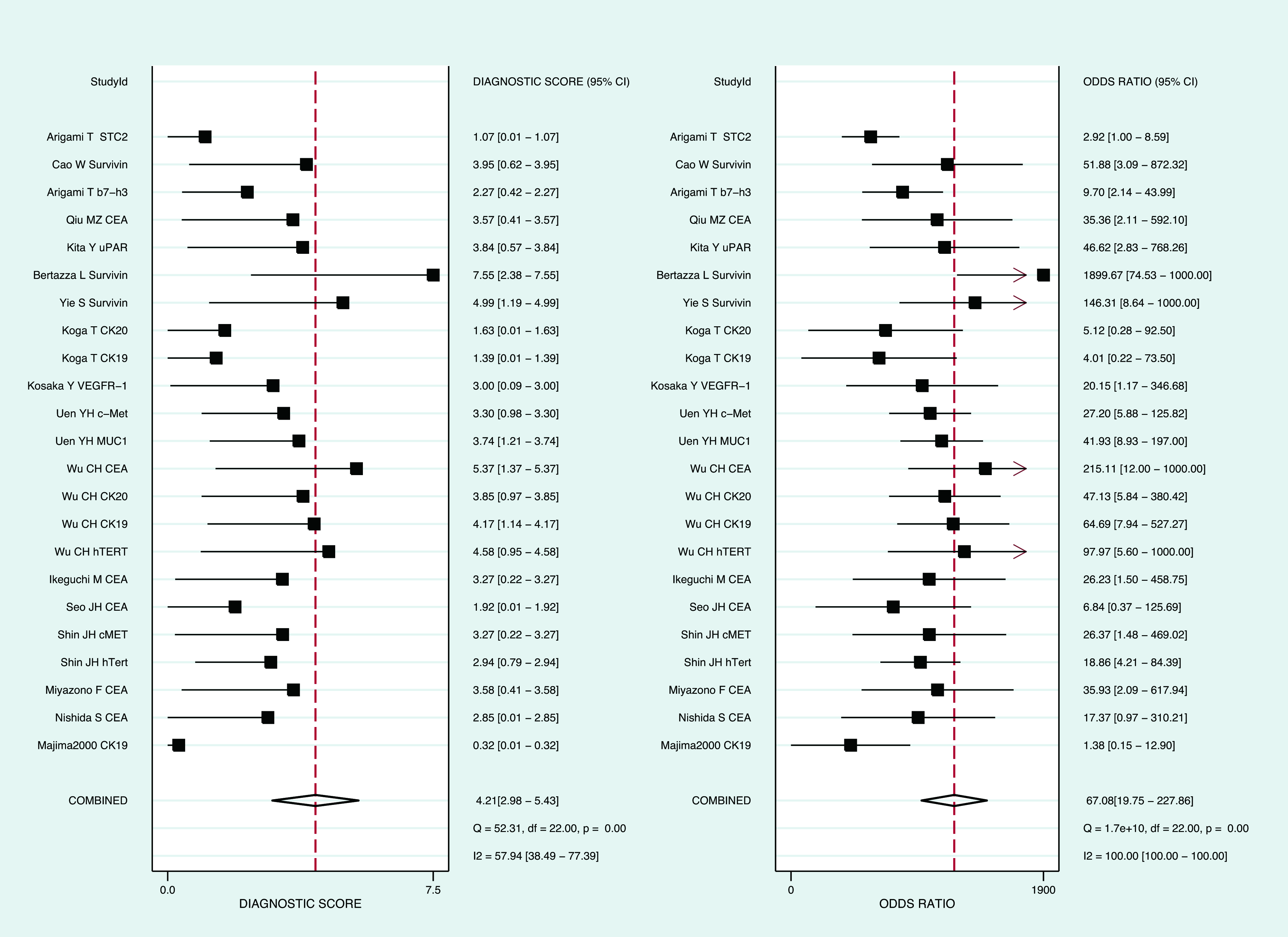

Supplement: Figure S3 — Forest Plot for pooled analysis of DOR. DOR, diagnostic odds ratio. (TIF) [file pone.0099259.s003.tif]

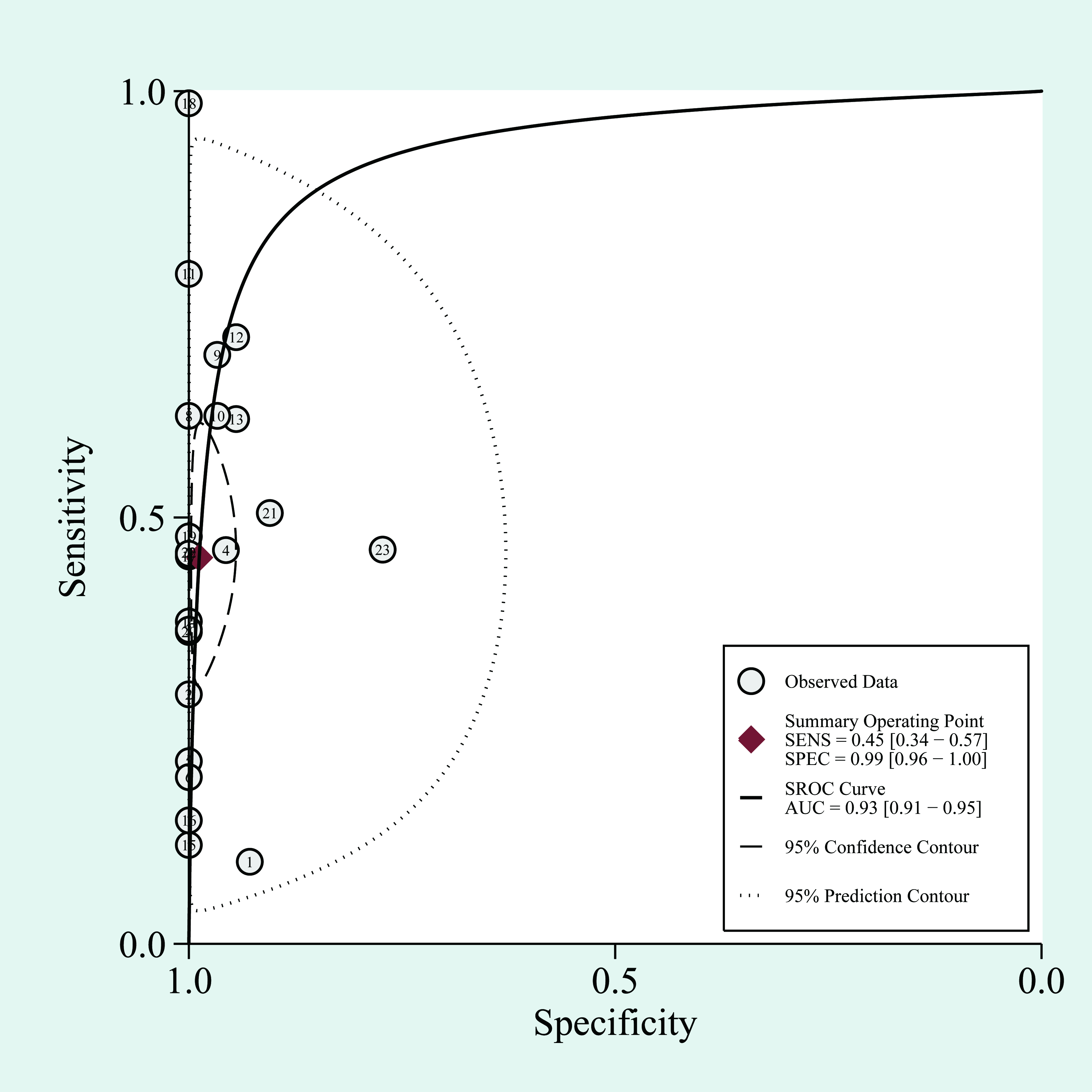

Supplement: Figure S4 — Summary ROC curve with confidence and prediction regions of sensitivity and specificity. (TIF) [file pone.0099259.s004.tif]

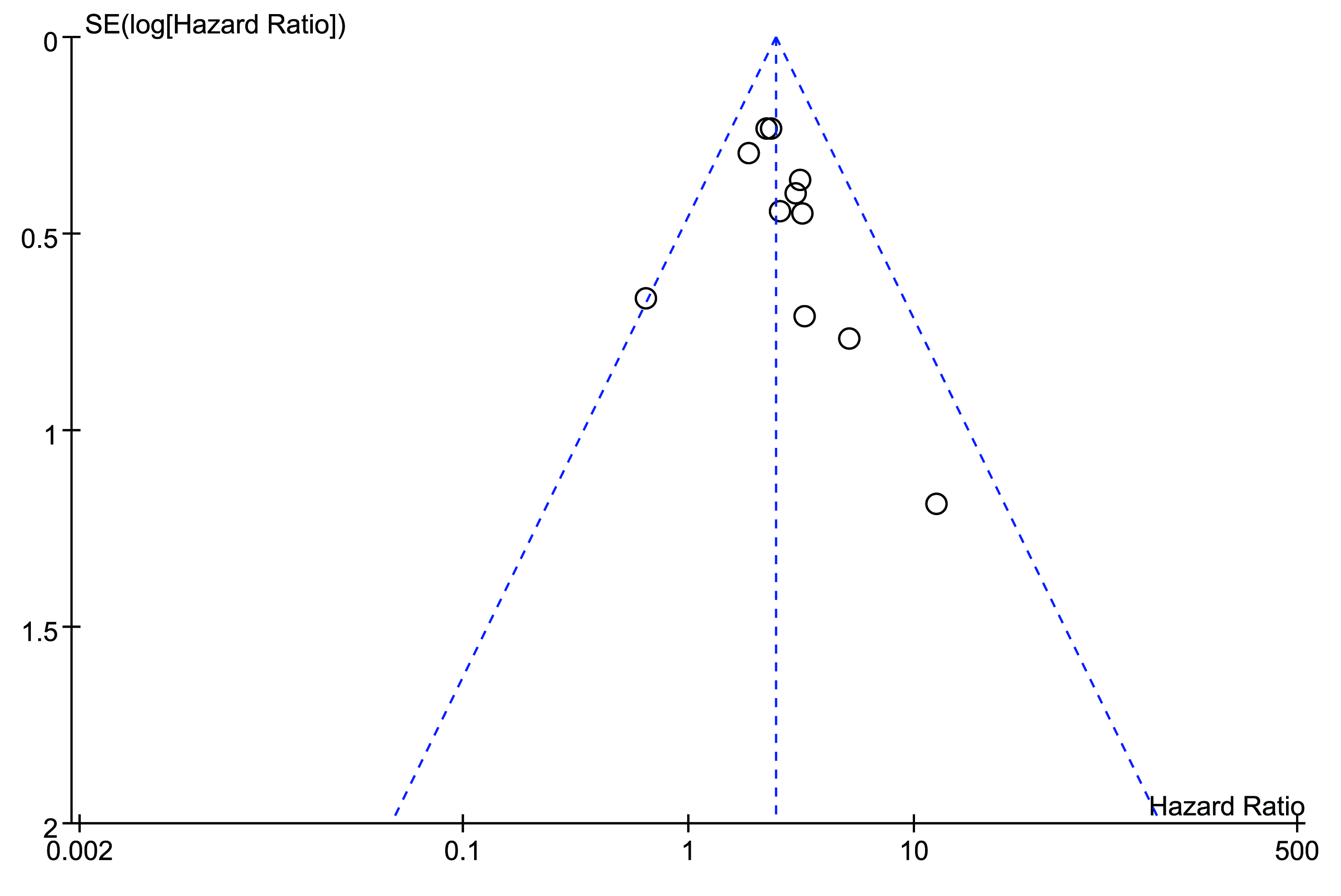

Supplement: Figure S5 — Funnel plot for summary estimates of RFS. RFS, relapse-free survival. (TIF) [file pone.0099259.s005.tif]

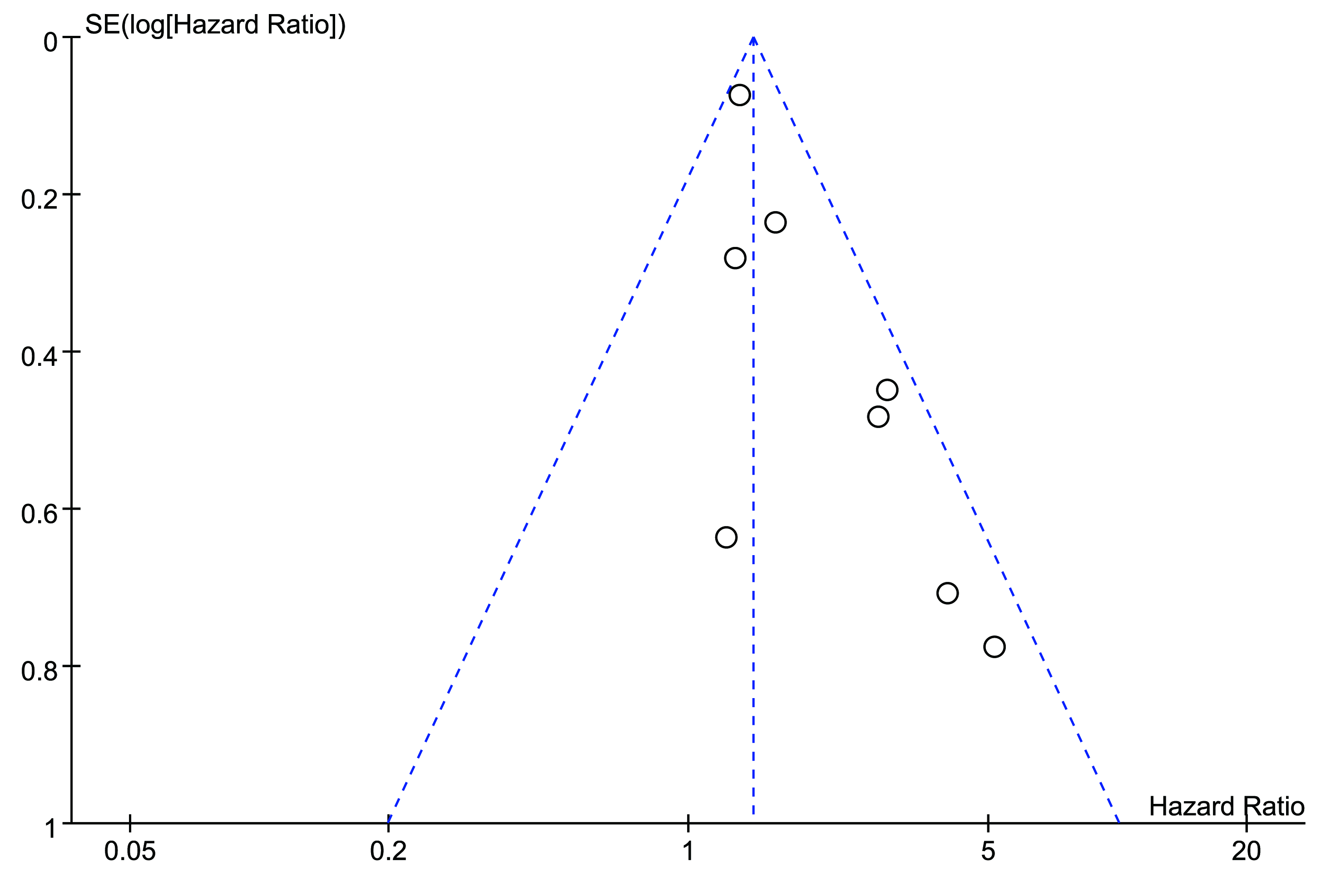

Supplement: Figure S6 — Funnel plot for summary estimates of OS. OS, overall survival. (TIF) [file pone.0099259.s006.tif]
